# When Less Is More: Volume Considerations in the Treatment of *Enterococcus faecalis* Endocarditis

**DOI:** 10.1093/ofid/ofag199

**Published:** 2026-05-20

**Authors:** Sami El-Dalati, William Harris

**Affiliations:** Division of Infectious Diseases, Department of Internal Medicine, University of Kentucky HealthCare, Lexington, Kentucky, USA; Department of Pharmacy Services, University of Kentucky HealthCare, Lexington, Kentucky, USA

**Keywords:** endocarditis, enterococcal infections, heart failure, oral antibiotic treatment

## Abstract

A 6-week regimen of intravenous ampicillin and ceftriaxone is currently the consensus, guideline-recommended, first-line treatment for *Enterococcus faecalis* endocarditis. This regimen is associated with significant salt and volume administration in a patient population susceptible to the development of hypervolemia and heart failure, which are associated with worse clinical outcomes. As a consequence of Hurricane Helene, the United States experienced significant intravenous fluid shortages in 2024 and 2025, prompting hospitals to switch from infusion delivery to push administration of many medications, without reported deleterious effects. Push administration of ampicillin and ceftriaxone and the evidence-based use of partial oral antibiotic therapy are treatment strategies that may help reduce the incidence of hypervolemia and/or heart failure in patients with *E faecalis* endocarditis.

A 61-year-old man with a history of obesity and a body mass index of 55 (calculated as weight in kilograms divided by height in meters squared) presented to the hospital after 3 weeks of fevers, night sweats, and 15-pound weight loss. *Enterococcus faecalis* grew in 2 sets of blood cultures. Transesophageal echocardiography revealed a 5-mm vegetation on the right coronary cusp of the aortic valve, with moderate aortic regurgitation and an ejection fraction of 60%–65%. Computed tomographic scans of the head, chest, abdomen, and pelvis were negative for embolic phenomena. Repeated blood cultures showed no growth at 5 days, and the cardiac surgeon recommended medical management given the lack of clear surgical indications and the patient's elevated surgical risk with a body mass index of >50 [1–3].

The infectious diseases consultant advised treatment with a 6-week course of intravenous ampicillin and ceftriaxone, and the patient was discharged to a nursing facility to complete a course of physical rehabilitation and antibiotic therapy. Three weeks later, he was readmitted with shortness of breath and lower-extremity edema. Chest radiography demonstrated bilateral opacities suggestive of pulmonary edema, the B-type natriuretic peptide level was markedly elevated, and transthoracic echocardiography demonstrated an unchanged 5-mm vegetation and stable moderate aortic regurgitation. Heart failure was diagnosed, and the patient was started on intravenous diuretics. Could his antibiotic regimen have contributed to this presentation?

## BACKGROUND

Enterococcal infectious endocarditis (IE) is a deep-seated infection that is difficult to treat. Ampicillin and ceftriaxone, when given together, have been shown to synergize, improving the activity of ampicillin [4]. Due to similar efficacy and improved tolerability compared with aminoglycoside-containing regimens, the 2015 American Heart Association and 2023 European Society of Cardiology (ESC) endocarditis guidelines recommend a combination of ampicillin and ceftriaxone for 6 weeks as first-line treatment of *E faecalis* IE [2, 3, 5]. The package insert for ampicillin recommends that it be diluted to ≤30 mg/mL of 0.9% saline for intravenous infusion, requiring ≥66.7 mL of fluid for a 2-g dose [6]. Practically, when administered at our institution in the inpatient or rehabilitation setting to individuals with normal renal function, this regimen requires patients to receive 2 g of ampicillin every 4 hours, with each dose coadministered with 100 mL of 0.9% saline and 2 g of ceftriaxone every 12 hours, also coadministered with 100 mL of 0.9% saline (Table 1). This translates to a daily administration of 800 mL of saline and 3787.6 mg of sodium. Even when administered in the outpatient setting using a continuous ampicillin infusion, due to stability concerns it is preferred for ampicillin to be coadministered with 600 mL of 0.9% saline [6].

**Table 1.** Estimated Sodium and Volume Burden of Ampicillin and Ceftriaxone Dosed for Treatment of *Enterococcus faecalis* Endocarditis at the Authors’ Institution

Administration Strategy	Antibiotic	Diluent	Total Daily Volume, mL^a^	Daily Sodium, mg			Total Daily Volume, mL; Sodium per Regimen, mg^b^
				Drug	Diluent	Total	
Intravenous infusion	Ampicillin (2 g every 4 h)	100 mL; 0.9% NaCl	600	789.6	2124	2913.6	800; 3787.6
	Ceftriaxone (2 g every 12 h)	100 mL; 0.9% NaCl	200	166	708	874	
Intravenous push	Ampicillin (2 g every 4 h)	18.8 mL; SWFI	112.8	789.6	0	789.6	151.2; 1091.5
	Ceftriaxone (2 g every 12 h)	19.2 mL; 0.9% NaCl	38.4	166	135.9	301.9	

Abbreviations: NaCl, sodium chloride; SWFI, sterile water for injection.

^a^Volumes calculated excluding overfill and drug powder volume.

^b^Ampicillin sodium includes 65.8 mg of sodium per 1 g of ampicillin; ceftriaxone sodium, 83 mg of sodium per 1 g of ceftriaxone; and 0.9% NaCl, 354 mg sodium of per 100 mL.

Approximately 19%–73% of patients with left-sided IE will experience heart failure and [3] virtually all national and international heart failure guidelines recommend that patients with heart failure restrict their sodium intake to between 1.5–3 g/d [7, 8]. The ampicillin/ceftriaxone regimen is also cumbersome for outpatient parenteral antibiotic therapy due to the multiple administration requirements and the frequent need for a double-lumen peripherally inserted central catheter (PICC), which is associated with higher rates of infection, occlusion, and thrombosis than single-lumen PICCs [9]. Patients with IE-related valvular regurgitation are at risk of heart failure, a class I indication for surgery by the American Heart Association and ESC guidelines [2, 3]. Even patients who undergo valve surgery remain at risk for postoperative hypervolemia, which can have a significant bearing on length of stay and rates of atrial fibrillation [10]. Notably, a 2023 analysis of antimicrobial treatment strategies for *E faecalis* endocarditis found a higher incidence of acute heart failure in patients treated with ampicillin and ceftriaxone compared to other regimens [11]. This raises the question whether, in certain cases of *E faecalis* IE, heart failure can be avoided with push delivery of ampicillin and ceftriaxone or by using an equally efficacious partial oral regimen.

## DISCUSSION

In 2024 and 2025, after Hurricane Helene devastated North Carolina and forced the closure of one of the largest drug manufacturing plants in the country, the United States experienced a 60% shortage in the availability of intravenous fluids [12]. Consequently, many hospitals converted appropriate antimicrobials, including ampicillin, to intravenous push formulations to conserve fluids. This strategy was similar to an approach adopted after Hurricane Maria in 2017, which was associated with decreased length of stay, less use of normal saline. and reduced costs without affecting mortality rates [13]. Ampicillin has typically not been given as intravenous push in adults due to a warning found in the package insert, which reads “Ampicillin for Injection, USP, 1 g or 2 g may also be given by direct intravenous administration… and administer slowly over at least 10–15 minutes. CAUTION: More rapid administration may result in convulsive seizures” [6]. Despite this, published data correlating ampicillin infusion rates with adverse events, including seizures, do not exist.

The change to intravenous push administration dramatically reduced the sodium and volume burden of ampicillin and ceftriaxone. For patients receiving treatment for *E faecalis* IE at our institution, the new administration reduced the total daily fluid administered from 800 to 151.2 mL and the sodium content from 3787.6 to 1091.5 mg (Table 1). The reduction could benefit patients with endocarditis and concomitant valvular insufficiency at risk for hypervolemia. There would be minimal impact in nursing time as the time required to give a push dose would be similar to the time required to connect and disconnect the 100 mL to an infusion pump. Furthermore, for months, ampicillin was given via push at our institution without reports of increased incidence of seizures. Review of internal data revealed that 837 patients received intravenous push administration of ampicillin during the shortage, and only 1 had a new seizure documented during their course of ampicillin; that patient had a known history of seizures and was already on antiepileptic medications.

Another potential strategy for avoiding hypervolemia in patients with *E faecalis* endocarditis is the use of partial oral antibiotic therapy after a period of intravenous therapy and clinical stabilization. In 2018, the POET trial evaluated 51 patients with *E faecalis* left-sided endocarditis who were transitioned to oral antibiotic regimens after ≥10 days of intravenous treatment and found no difference in a composite outcome of mortality, relapsed bacteremia, need for unplanned valve surgery, or embolic events when comparing these patients with others who received full courses of intravenous antibiotics [14]. A subsequent clinical implementation study by the same investigators reported similar outcomes for another 45 patients with *E faecalis* endocarditis treated with partial oral antibiotics [15]. However, neither article reported the rates of hypervolemia or new heart failure symptoms during therapy. Our own study reported similar findings at 90 days for 20 patients with *E faecalis* endocarditis treated with partial oral therapy antibiotics [16], and the 2023 ESC guidelines include recommendations for the use of oral antibiotics [3]. Despite these data and these recommendations, adoption of partial oral antibiotic treatment has been slow; a 2025 survey of infectious disease physicians found that approximately half never transition patients with IE to oral therapy with even lower rates of use for patients with *E faecalis* [17].

## CONCLUSIONS

While the proportion of patients with *E faecalis* IE who experience complications of hypervolemia or heart failure while on therapy with ampicillin and ceftriaxone is not currently known, we regularly experience volume-related challenges in this population. As the fluid shortage resolves and hospitals transition back to intravenous infusion administration of antimicrobials, maintaining the option for push-dose ampicillin and ceftriaxone may be beneficial for certain patients with *E faecalis* endocarditis, with decisions made on a case-by-case basis, ideally with the input of a multidisciplinary endocarditis team [3].

Consideration of intravenous push administration of these antimicrobials during the initial “critical phase” of treatment (the first 10 days after blood culture clearance) would reduce the salt and volume burden for patients [3]. Providers could continue with the push regimen in the “continuation phase” (after 10 days of initial therapy) or consider transitioning to an evidence-based oral antibiotic regimen to further reduce salt and volume administration and avoid the challenges of outpatient parenteral antibiotic therapy [3].
